# Cardiac Hypertrophy: From Pathophysiological Mechanisms to Heart Failure Development

**DOI:** 10.31083/j.rcm2305165

**Published:** 2022-05-06

**Authors:** Alfredo Caturano, Erica Vetrano, Raffaele Galiero, Teresa Salvatore, Giovanni Docimo, Raffaella Epifani, Maria Alfano, Celestino Sardu, Raffaele Marfella, Luca Rinaldi, Ferdinando Carlo Sasso

**Affiliations:** ^1^Department of Advanced Medical and Surgical Sciences, University of Campania “Luigi Vanvitelli”, I-80138 Naples, Italy; ^2^Department of Precision Medicine, University of Campania “Luigi Vanvitelli”, I-80138 Naples, Italy

**Keywords:** cardiac hypertrophy, heart failure, pathophysiology, diabetic cardiomyopathy, metabolism

## Abstract

Cardiac hypertrophy develops in response to increased workload to reduce ventricular wall stress and maintain function and efficiency. Pathological hypertrophy can be adaptive at the beginning. However, if the stimulus persists, it may progress to ventricular chamber dilatation, contractile dysfunction, and heart failure, resulting in poorer outcome and increased social burden. The main pathophysiological mechanisms of pathological hypertrophy are cell death, fibrosis, mitochondrial dysfunction, dysregulation of ${\mathrm{Ca}}^{2+}$-handling proteins, metabolic changes, fetal gene expression reactivation, impaired protein and mitochondrial quality control, altered sarcomere structure, and inadequate angiogenesis. Diabetic cardiomyopathy is a condition in which cardiac pathological hypertrophy mainly develop due to insulin resistance and subsequent hyperglycaemia, associated with altered fatty acid metabolism, altered calcium homeostasis and inflammation. In this review, we summarize the underlying molecular mechanisms of pathological hypertrophy development and progression, which can be applied in the development of future novel therapeutic strategies in both reversal and prevention.

## 1. Introduction

The main function of the heart is to sustain peripheral organ perfusion in both normal and stress conditions. To achieve this goal, cardiac tissue exhibits plasticity that allows the heart to respond to environmental demands [1]. Cardiac hypertrophy (CH) mainly develops in response to an increased preload and/or afterload to preserve cardiac output, and rarely due to genetic mutations or exposure to growth factors. In case of increased workload, heart hypertrophy determines an increased contractility, at least at the beginning, a decrease in left ventricular wall stress due to increased wall thickness (Laplace’s law), and changes in gene expression with a consequent modification in heart metabolism, contractility, and cardiomyocytes survival. CH can be divided into physiological and pathological. Both conditions share cardiomyocytes enlargement and are a response to cardiac stress [1].

Cardiac growth during ontogenetic development, instead, is characterized by both hyperplastic and hypertrophic growth and is not considered CH [2]. Physiological CH is characterized by a mild increase in cardiac mass (10–20%), mainly due to cardiomyocytes’ hypertrophy in response to body growth or exercise, with an adequate capillary network expansion to provide appropriate cardiomyocyte nourishment. In this setting, no structural or functional cardiac abnormalities can be detected, and physiological hypertrophy is generally not considered to be a risk factor for heart failure (HF) [2]. In fact, heart contractility is preserved or increased in absence of interstitial or replacement fibrosis or cell death. Moreover, physiological hypertrophy is fully reversible. However, it should also be recognized that in case physiological hypertrophy becomes quantitatively excessive or sustained for a prolonged period, it can evolve into maladaptive. Pathological hypertrophy, instead, can be adaptive at the beginning with concentric growth of the ventricle as compensatory response, and is reversable if the primary stimulus is reversed before the development of intrinsic disease. If the stimulus is not solved, physiological hypertrophy progresses to ventricular chamber dilatation with wall thinning through lengthening of individual cardiomyocytes, contractile dysfunction, and, finally, HF with both preserved and reduced ejection fraction, arrhythmias, and death [3]. Aim of this review is to provide an insight into the most updated main pathophysiological mechanism of CH and consequent HF development.

## 2. Mechanisms of Pathological Cardiac Hypertrophy 

**Fig. 1.**
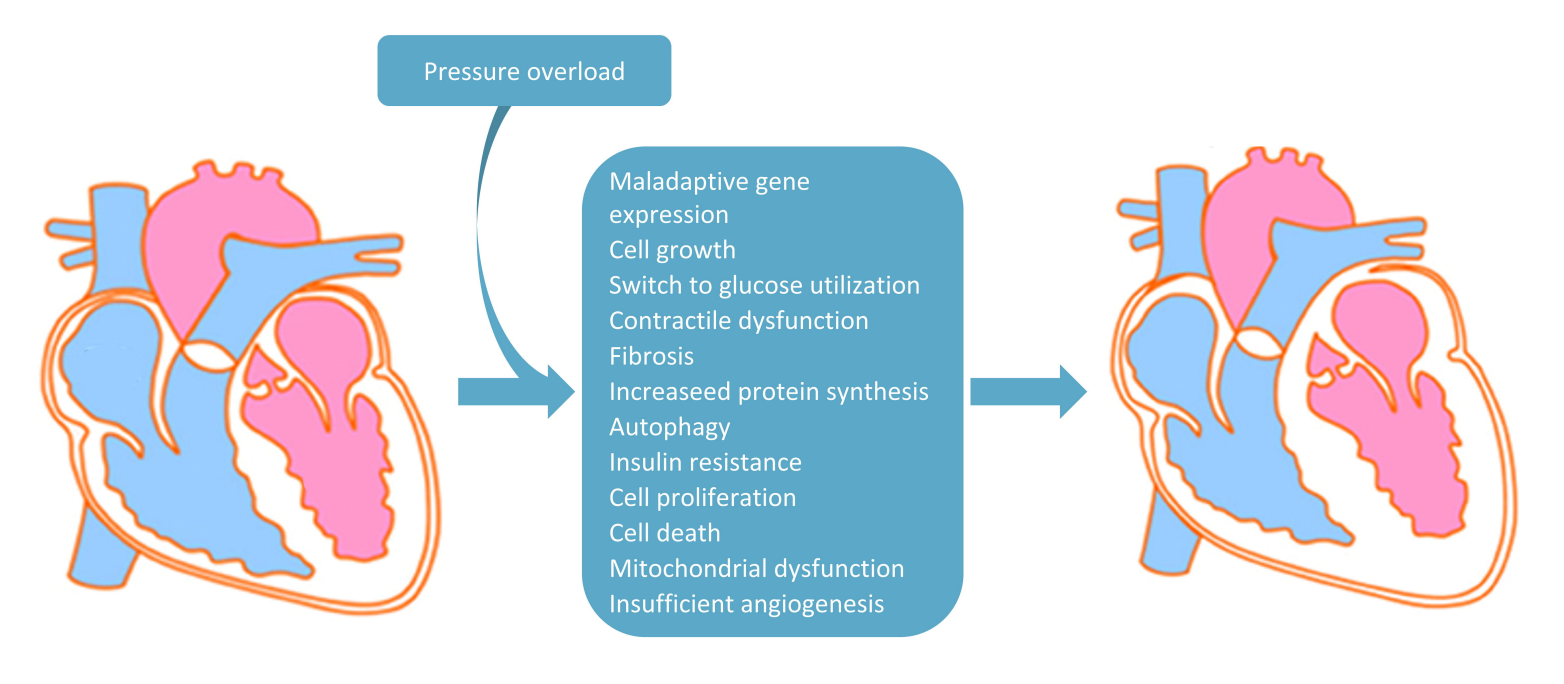
**Main pathophysiological changes due to pressure overload inducing cardiac hypertrophy**. In the diagram are listed all the main pressure overload-induced pathophysiological mechanisms involved in the shift from non-hypertrophic (on the left) to hypertrophic heart (on the right).

Cardiac disfunction due to hypertrophy develops when, beyond cell growth and protein synthesis, following further processes are established: cell death, fibrosis, mitochondrial dysfunction, dysregulation of ${\mathrm{Ca}}^{2+}$-handling proteins, metabolic changes, fetal gene expression reactivation, impaired protein and mitochondrial quality control, altered sarcomere structure, and inadequate angiogenesis (Fig. 1) [1, 2, 3]. Pathological conditions, such as hypertension and myocardial infarction, can induce pathological hypertrophy development mostly due to neuroendocrine hormones activation and heart overload, also showing different downstream signalling pathways from the one presented by physiological hypertrophy [1].

### 2.1 Angiogenesis

Myocardial angiogenesis plays a key role in maintaining an adequate nutrient supply, to avoid myocardial disfunction onset [4, 5]. Moreover, angiogenesis stimulation during pressure-overload (PO) can prevent the switch from compensatory CH to HF [6]. Furthermore, in patients with aortic stenosis and preserved ejection fraction it was found a strong correlation between myocardial blood vessel density and left ventricular mass index [7]. On the other hand, a significant reduction in myocardial capillary density was associated with advanced pathological remodelling and HF [8, 9]. Hypoxia-inducible factor 1α (HIF1α) is one of the major transcription factors that regulates oxygen homeostasis through angiogenesis, vascular remodelling, and glucose metabolism [10]. When stimulated, it induced HIF1α-responsive angiogenic factors production, including Vascular Endothelial Growth Factor (VEGF), which in turn promote cardiomyocyte growth and angiogenesis. However, in pathological hypertrophy, the cellular tumour antigen p53 is upregulated, thus promoting ubiquitylation and proteasomal degradation of HIF1α, probably through the E3 ubiquitin-protein ligase MDM2, which in turn leads to an unbalance between myocardial growth and capillary density, thereby promoting maladaptive CH and he HF onset [11, 12].

### 2.2 Angiotensin II and Endothelin 1 

Angiotensin II and endothelin 1 are peptide hormones that bind to the G protein-coupled angiotensin II receptor and endothelin 1 receptor, respectively, resulting in the activation of G proteins such as $G_{q/11}$, which in turn leads to diacylglycerol (DAG) and inositol trisphosphate (IP3) synthesis. In the Golgi apparatus DAG activates nuclear protein kinase D and CH [13]. IP3, instead, promotes the release of intracellular ${\mathrm{Ca}}^{2+}$ from the endoplasmic and sarcoplasmic reticulum, which in turn activates the ${\mathrm{Ca}}^{2+}$–calmodulin complex and calcineurin. The ${\mathrm{Ca}}^{2+}$–calmodulin complex activates protein kinase Cα (PKCα) and the serine/threonine kinase calcium/calmodulin-dependent protein kinase type II (CaMKII). PKCα regulate a cascade pathway which is involved in the cardiac contractility [14]. In fact, PKCα knock-out mice shows cardiac increased contractility and are more resistant to PO-induced HF, while cardiac-specific *Prkca* overexpression promotes contractile dysfunction [15]. CaMKII isoform δ is the most prevalent in the heart, and, in case of PO, it enhances the progression to maladaptive pathological hypertrophy and HF by activating ryanodine receptor 2, which in turn mediate ${\mathrm{Ca}}^{2+}$ leak from the sarcoplasmic reticulum. CaMKII is not only activated by ${\mathrm{Ca}}^{2+}$–calmodulin complex, but also by exchange proteins directly activated by cAMP (EPACs) and by reactive oxygen species [16, 17]. Oxidative stress is also involved in the downregulation of nucleocytoplasmic shuttling HDAC4, a class II histones which plays an important role as a repressor of pathological hypertrophy, in contrast with class I histones (HDAC1, HDAC2 and HDAC3) [18, 19, 20, 21].

Calcineurin is a ${\mathrm{Ca}}^{2+}$-activated serine/threonine protein phosphatase which interacts with nuclear factor of activated T cells (NFAT), thus promoting NFAT nuclear localization to modulate pathological pro hypertrophy gene transcription [21, 22]. This modulation is mediated by NFAT interaction with transcriptional cofactors (GATA4 and myocyte-specific enhancer factor 2A (MEF2A)). Recent studies have hypothesized another pathway for the activation of calcineurin/NFAT signalling, through the activation of transient receptor potential cation (TRPC) channels. TRPC channels are involved in the regulation of ${\mathrm{Ca}}^{2+}$ and ${\mathrm{Na}}^{+}$ movement in specific microdomains, and it seems that this channel expression and activation is increased in pathological hypertrophy or HF [23, 24, 25, 26]. Among all TRPC isoforms, the most studied are TRPC1 and TRPC3. It has been reported that inhibiting this isoforms gene expression ameliorate CH induced by agonists and PO [23, 27].

### 2.3 Catecholamines

Increased sympathetic nerve activity produces an increased concentration of blood catecholamine, which in turn is related to a poor prognosis in patients affected by HF [28]. Moreover, high adrenergic activity is associated with CH and the basal plasma levels can also predict CH extent in patients affected by hypertension, independently of systolic blood pressure and body mass index [29]. The role played by β-blockers use to reverse β-adrenergic receptor desensitization has been assessed by several clinical trials [30, 31]. Catecholamines are neuroendocrine hormones that thorough the binding of both α-adrenergic and β-adrenergic receptors, can activate adenylyl cyclase to increase cAMP levels, which in turn activates protein kinase A (PKA), leading to an increase in in cytosolic ${\mathrm{Ca}}^{2+}$ levels. In several diseases, such as hypertension, myocardial infarction, and HF, the chronic β-adrenergic stimulation is responsible for pathological hypertrophy and receptor desensitization development, through GPCR kinase (GRK)-mediated β-arrestin signalling modulation [32]. GRK2 and GRK5 are the most expressed in the heart and seems to be upregulated in patients affected by HF [33, 34]. In mice model, in which pathological CH was induced through transverse aortic constriction (TAC), it was found that overexpression of GRK5 was able to worsen pathological hypertrophy through HDAC5 or NFAT signalling modulation [35, 36]. In contrast, cardiac-specific GRK5-knockout mice have revealed a reduced hypertrophy and maladaptation response to PO compared to wild phenotype mice [37]. cAMP can also directly activate EPAC1, which is mostly expressed in the heart and can be upregulated by both PO and HF, due to β-adrenergic receptor stimulation. In this setting, EPAC1 overexpression can induce pathological hypertrophy development through a PKA independent activation of the calcineurin–NFAT and the CaMKII–MEF2A pathways [38, 39]. In contrast, in mice TAC-induced CH model, EPAC1 downregulation was not associated with improved hypertrophy, but was associated with prevention of both transition to maladaptive remodelling and HF [40, 41].

Finally, catecholamines are also implied in another pathway which leads to CH, through G protein MAPK cascade activation. In fact, MAPK activate both MEK3/MEK6 and MEK4/MEK7, which in turn activate p38 kinases and JUN N-terminal kinases (JNKs), respectively, which ends with GATA4-mediated pathological hypertrophy gene transcription [42].

### 2.4 mTOR Signalling 

Increased protein synthesis or decreased degradation is necessary for cardiomyocyte cell growth. In this setting a key role is played by mTOR (mammalian Target Of Rapamycin) signalling cascade, which is involved in the modulation of growth factors signalling and amino acids availability, of cell metabolism and growth. mTOR is a serine/threonine-protein kinase that works as part of two distinct complexes, mTORC1 and mTORC2 [43]. mTORC1 activity is increased in both physiological and pathological hypertrophy and represent an essential adaptive mechanism during acute PO [44]. However, mTORC1 sustained activation can be harmful as it may lead to autophagy suppression with consequent protein quality control deterioration. mTORC1 induce ribosomal protein production through a direct activation of ribosomal protein S6 kinaseβ1 (S6K1) and inhibition of eIF4E-binding protein 1 (4EBP1) [45]. However, it seems that S6K1 may possibly not be involved in mTORC1 pro-hypertrophic effects [46, 47]. In fact, only a modest CH was detected in cardiac-specific overexpression of the gene encoding S6K1 (*Rps6kb1*), while no association was found for overexpression of the gene encoding S6K2 (*Rps6kb2*) [47]. Moreover, in a* Rps6kb1*, *Rps6kb2*, or both knock-out mice model, no difference was reported on CH induced by exercise, PO, or IGF1R–PI3K signalling [47]. 4EBP1 seems to play a crucial role in mTORC1-induced CH, though more studies are needed [44]. mTORC2 is also stimulated during PO and inhibits cell death by downregulating pro-apoptotic mammalian STE20-like protein kinase 1, showing an opposite role compared to mTORC1 [48].

### 2.5 Insulin, Insulin Receptor and Akt Signalling 

Insulin is an anabolic hormone involved in cardiac physiological hypertrophy. Growing evidence have suggested that insulin, insulin receptor activation and following Akt signalling are beneficial when maintained in physiological range [49]. In fact, insulin signal suppression leads to physiological hypertrophy inhibition, whereas overstimulation results in pathological CH and disturbs homeostasis induction. It seems that chronic hyperinsulinemia can promote these pathological changings through the activation of angiotensin II signalling, and intensive glycaemic lowering by insulin has also been associated with increased cardiovascular events and future risk of HF [49, 50]. In chronic PO murine model was reported an increase in cardiac insulin signalling with a mismatch development of cardiomyocytes size and vascularity, leading to myocardial hypoxia development, thus resulting in cardiomyocytes death and cardiac dysfunction. On the other hand, cardiomyocyte hypertrophy and cardiac dysfunction due to PO were significantly reduced in heart heterozygous insulin receptor deletion or Akt deletion [51]. Moreover, Akt-mTOR signalling was reported to be reduced in CH murine model, whereas it was found increased in physiological hypertrophy, making it easy to suggest its implication in the type of hypertrophy differentiation [52]. Insulin resistance prevalence is increased in patients affected by systolic dysfunction and is accompanied by an enhanced risk of HF development. It has been hypothesized a possible link between metabolic disfunction and HF, though the underlying mechanisms are still obscure [53, 54, 55, 56, 57].

### 2.6 Natriuretic Peptides

Atrial natriuretic peptide (ANP) and brain natriuretic peptide (BNP) are both secreted by cardiomyocytes and can inhibit CH by activating cyclic GMP-dependent protein kinase (PKG) signalling [58, 59]. Cardiac-specific deletion of the ANP receptor 1 (NPR1) encoding gene can develop mild hypertrophy that worsen with PO stimulus, which can in turn evolve into pathological hypertrophy and cardiac remodelling [60]. PKG signalling activation, due to natriuretic peptide binding with their receptor, leads to inhibition of calcineurin–NFAT, TRPCs, and RHOA–RHO kinase pathways [61]. cGMP can be degraded by cGMP specific $3^{\prime }$,$5^{\prime }$-cyclic phosphodiesterase 5A or 9A, thus antagonizing NO and natriuretic peptide action. Moreover, animal and human models have reported an increased activity of both PDE5A and PDE9A in pathological hypertrophic heart [62, 63]. Therefore, genetic or pharmacological inhibition of PDE9A was found to attenuate pathological responses to both neurohormones and sustained PO, independently of the NO pathway [63].

### 2.7 Non-Myocytes and Immune Cells 

Fibroblasts are one of the most prevalent cell types in cardiac tissue and crosstalk with other heart’s cells, such as immune and endothelial cells, and cardiomyocytes [64, 65, 66]. Their role is to contribute to homeostasis maintenance and tissue remodelling in response to stress. Cardiac fibroblasts express extracellular matrix (ECM) receptors coupling mechanical stimuli to functional responses, with the aim of adapting the composition and stiffness of the ECM and fibrotic response to mechanical stress [66, 67]. Their involvement in CH is not well defined, and target genes depletion study resulted nonspecific. However, fibroblasts manage to communicate with cardiomyocytes by secreting humoral mediators such as growth factors, including fibroblast growth factors (FGFs), transforming growth factor-β1 (TGFβ1), and IGF1 which are implied in cardiomyocyte growth, death, and cardiac fibrosis development [68]. In mice model FGF2 deletion is implied in dilated cardiomyopathy development in absence of compensatory hypertrophy in response to angiotensin II stimulation or PO, probably due to the lack of activation of JNK and p38 MAPK pathways [69, 70]. TGFβ, instead, is upregulated in response to pathological stimuli and according to the canonical TGFβ–SMAD2/SMAD3 signalling or noncanonical SMAD–TAK1 signalling activation it can lead to cardiac fibrosis without hypertrophy, or pathological hypertrophy and fibrosis, respectively [71, 72, 73, 74]. Fibroblast expression of Interleukin-11 has been recently identified as a crucial downstream effector of TGFβ. In fact, genetic deletion of *Il11ra* has shown a cardiac fibrosis inhibition without affecting hypertrophy, thus underlying its main role in cardiac fibrosis and contractile dysfunction development [75]. Fibroblast secreted IGF1 have proven to suppress PO-induced CH [76]. In a murine model IGF1r overexpression was associated with physiological hypertrophy gene transcription promotion [77]. Cardiac fibroblast can also secrete miRNA-enriched exosomes, such as miR-21-3p which seems to be involved in pathological hypertrophy development as a result of cardiomyocyte suppression of Sorbs2 and Pdlim5 [78]. Therefore, numerous cytokines, produced by fibroblasts, and by both resident and circulating immune cells have proven to be correlated with hypertrophy and HF [79].

Rodent model studies have underlined the role of inflammation in HF, suggesting its unfavourable impact on cardiac homeostasis [80, 81, 82, 83], also showing a correlation with pro-inflammatory cytokines blood levels, such as tumor necrosis factor (TNF)-α, IL-6, and IL-1β, and HF severity [84]. In murine cardiomyocyte-specific TNF-α overexpression models an increased CH and fibrosis was reported, leading to cardiac dysfunction [85]. By contrast, TNF-α deletion was associated with CH and remodelling improvement [86]. IL-6 interacts with IL-6 receptor subunit-β (IL-6Rβ) and activates the JNK pathway. In murine model, it was reported that infusion of IL-6 or activation of IL-6Rβ was associated with pathological hypertrophy development, whilst, IL-6 deletion inhibits TAC-induced hypertrophy, probably due to suppression of CaMKII dependent STAT3 pathway [87, 88, 89]. However, gene encoding IL-6Rβ depletion is responsible for an acute maladaptive remodelling and HF onset in response to PO [90]. It was reported that human IL-1a overexpression in mice stimulates CH with preserved contractile function [91]. IL-1b-deficient mice with PO, instead, show an increased contractile dysfunction with reduced hypertrophy and fibrosis, mostly due to insufficient IGF1 (JAK–STAT-mediated) production in cardiac fibroblasts [92]. Anakinra, an IL-1 receptor antagonist, has proven to ameliorate exercise tolerance in patients with recent episode of decompensated systolic HF and in HF with preserved ejection fraction (HFpEF) patients [93, 94]. In patients with anamnesis of myocardial infarction and a high inflammatory burden, canakinumab, a monoclonal antibody targeting IL-1β, have proven to lower cardiovascular events recurrence, independently of lowering LDL-cholesterol levels [95]. IL-10 is an anti-inflammatory cytokine, and it was reported to suppress PO inducing CH through nuclear factor-κB (NF-κB) signalling inhibition. IL-10 knockout mice with isoprenaline-induced and TAC-induced CH can exacerbate cardiac maladaptive remodelling, while IL-10 supplementation prevents or even reverses TAC-induced cardiac remodelling by activating STAT3 and inhibiting NF-κB [96]. Increased IL-10 production also modulates cardiac maladaptive remodelling due to α-galactosylceramide natural killer T cells activation and inhibition of T cell immune activity with abatacept [97, 98].

Endothelial cells modulate the cardiomyocyte growth through paracrine regulation [99]. In response to PO, endothelial cells secrete IL-33, which binds to membrane-bound ST2 (IL-1RL1) in cardiomyocytes [100]. IL-33 or IL-1RL1 deletion is associated with increased PO hypertrophy, while recombinant IL-33 infusion ameliorates it, together with fibrosis, due to NF-κB activation [100, 101]. In both human and animal model of CH, a higher endothelial cells production of complement C1q/TNF-related protein 9 (CTRP9) was detected. Moreover, in mice model of CTRP9 deletion, TAC-induced CH and dysfunction was decreased as a result of the reduced activation of the MAPK7–GATA4 signalling pathway [102]. As CTRP9 supplementation ameliorates myocardial infarction cardiac remodelling through PKA-dependent pathway, it has been hypothesized a CTRP9 stress-dependent pathological hypertrophy regulation [103].

### 2.8 Mechano-Sensors 

#### 2.8.1 Canonical Transient Receptor Potential Channels

TRPCs are a family of nonselective cation channels that can control pathological hypertrophy development mainly through calcineurin and NFAT signalling effectors [104]. TRPC3 and TRPC6 promote pathological hypertrophy development through calcineurin-dependent signalling, which is inhibited by their depletion in murine model [105, 106]. PKG phosphorylation of both TRPC3 and TRPC6, instead, is implied in channel conductance reduction, thus inhibiting TRPC-mediated hypertrophy [107]. TRPC3, TRPC4, and TRPC6, dominant negative gene variant overexpression was found to be protective against PO pathological hypertrophy development [27]. In myocardial infarction TRPC4 knockdown mouse model it was reported an improvement in pathological hypertrophy, cardiac performance, progression to HF, and increased survival when compared to wild type animals [108].

#### 2.8.2 Stromal Interaction Molecule 1

Stromal interaction molecule 1 (STIM1) is a ${\mathrm{Ca}}^{2+}$ sensor which allows ${\mathrm{Ca}}^{2+}$ entry in response to endoplasmic reticulum ${\mathrm{Ca}}^{2+}$ store depletion by coupling with ${\mathrm{Ca}}^{2+}$-release-activated ${\mathrm{Ca}}^{2+}$ channel protein 1. In murine TAC-induced model, STIM1 is upregulated and enhance a mechanism known as store-operated ${\mathrm{Ca}}^{2+}$ entry by activating NFAT and CaMKII signalling pathways, therefore promoting both pathological hypertrophy and arrhythmias onset [109, 110]. STIM1 *in vitro* deletion was associated with agonist-induced hypertrophy inhibition, whereas STIM1 silencing *in vivo* prevents the PO-induced hypertrophy development but can also reverse preestablished CH, though burdened by a rapid HF onset, mainly through mTORC2–AKT–GSK3β pathway [110, 111]. It is thought that STIM1 by increasing ${\mathrm{Ca}}^{2+}$ flux during the initial phase of CH may induce adaptive hypertrophy, which in chronic phase become pathological.

### 2.9 Epigenetic Modifications

Epigenetic modifications regulate chromatin structure, thus controlling gene expression by filtering promoters and enhancers access to DNA. In particular, histone acetylation on lysine residues promotes chromatin relaxation, with enhanced transcriptional activation, whereas histone acetylation suppression is implicated in chromatin condensation and gene expression inhibition. Therefore, epigenetic modifications can induce pathological hypertrophy onset by modulating genome architecture and stability, and gene expression. It was reported that trimethylation of histone H3 at lysine 4, 9, or 27 and demethylation of H3 at lysine 9 and 79 modulate a pro pathological hypertrophy gene expression [112]. Histone lysine-specific demethylase 4A (KDM4A) is increased during pathological hypertrophy, leading to FHL1 enhanced expression that further promotes hypertrophy and HF development [113]. Histone acetylation is modulated by histone acetyltransferases (HATs) and histone deacetylases (HDACs). In mice, HATs overactivation induces CH and left ventricular remodelling [114, 115]. HDACs, instead, are divided into three classes: class I (HDAC1, 2, 3, and 8), class II (HDAC4, 5, 6, 7, 9, and 10), and class III. Class I HDACs mediate cardiac hypertrophic responses, with HDAC2 acting as an indirect down regulator of Akt/GSK-3β pathway [116]. Class II HDACs seems to play an anti-hypertrophic role. In fact, in mice, HDAC5 or HDAC9 depletion is linked to CH enhancement by silencing MEF2C [117, 118]. Class III HDACs are associated with CH inhibition and increased cardiomyocyte survival.

MicroRNA (miRNA) is a class of small non-coding RNAs involved in translation repression or transcriptional degradation of target mRNAs [119]. A single miRNA may interact with multiple target genes, making it easy to suggest a possible role in CH development, and HF, mostly due to MiRNAs targeting mRNAs encoding ${\mathrm{Ca}}^{2+}$-handling proteins and proteins involved in ${\mathrm{Ca}}^{2+}$-responsive signalling pathways [120, 121]. It was reported that pathological stimuli induce miRNA-217 downregulate mRNA *EHMT1* and *EHMT2*, and histone-lysine N-methyltransferases EHMT1 and EHMT2 inhibition is involved in pathological hypertrophy development, though EHMT2 role is still controversial [122, 123, 124]. miRNA-212 and miRNA-132 were also associated with pathological hypertrophy by FoxO3 expression inhibition and calcineurin/NFAT signalling and autophagy suppression upregulation [125].

Moreover, the long non-coding RNA (lncRNA) CH-associated epigenetic regulator (Chaer) is involved in pathological hypertrophy development, by interacting with Polycomb repressor complex 2 (PRC2) in response to hypertrophic stimuli [126]. This interaction leads to the inhibition of H3 lysine 27 methylation in the promoter regions of fetal genes related to CH, such as *Acta1*, *Anf*, and *Myh7* in mice, thus developing pathological hypertrophy [42].

### 2.10 Time is the Key

Most of the aforementioned signalling mechanisms contributing to pathological hypertrophy are initially activated as an adaptive response. However, sustained activation of these signalling mechanisms has a major role in inducing cardiac pathological hypertrophy and HF development. In fact, while short-term AKT activation promotes cardiomyocytes physiological growth, sustained activation of AKT has been associated with pathological hypertrophy and HF development [42]. Finally, the functional consequences of each stimulus and cardiomyocytes response depend on the balance between cardioprotective and detrimental effects.

## 3. Metabolic Changes

The heart consumes a huge amount of ATP to perform its action. Impaired energy metabolism adaptation during hypertrophic response enhances pathological hypertrophy and cardiomyocyte death, thus preceding HF development [127, 128]. About 70–90% of the physiological heart ATP production derives from fatty acids (FA) oxidative phosphorylation, whilst 10–30% derives from glucose, lactate, and ketone bodies oxidation [129]. During pathological hypertrophy and HF development a metabolic impairment leads to a shift in cardiac energy production from FA to glycolysis, anaplerosis, and other forms of metabolism [130, 131, 132]. This metabolic reprogramming is associated with mitochondrial energy transduction and respiratory pathways downregulation, and it begins during hypertrophy early stages [133]. However, changes in FA ATP production are more consistent than glycolysis and of the other metabolic substrates, thus causing a progressive reduction of ATP synthesis, which in turn leads to energy deficiency and HF development [129, 134]. In murine model it was reported that *Acacb *deletion, which encodes in acetyl coenzyme A carboxylase 2, enhance FA oxidation, thus ameliorating both pathological hypertrophy and HF development by preserving the substrate utilization profile, making it easy to suggest that metabolic reprogramming might be a direct cause of pathological hypertrophy [134, 135].

In the heart, the nuclear receptors peroxisome proliferator activated receptor-α (PPARα) and PPARγ only regulate FA metabolism, whilst PPARβ and PPARδ are involved in both FA and glucose metabolism regulation [136]. In addition, ERRα also regulates FA metabolism as well as stimulating mitochondrial oxidative phosphorylation gene expression [137, 138]. PPARs and ERRs are in turn modulated by transcriptional cofactors PGC1 family. In the heart, PGC1α and PPARα are upregulated due to exercise and are downregulated during pathological conditions [139, 140]. Altered cellular metabolism occurs in response to chronically altered workload and substrate availability, with consequent decreased energy production and increased oxidative stress, resulting in cardiomyocyte death and fibrosis, leading to maladaptive hypertrophy and HF.

### 3.1 Glucose Metabolism

Glucose is carried into cellular cytoplasm through glucose transporters (GLUTs). In particular, GLUT1 is most expressed in fetal heart, whereas GLUT4 in the adult one. However, in pathological hypertrophy, insulin-independent HepG2 glucose transporter GLUT1 levels are increased, whereas GLUT4 levels are reduced, together with an enhanced glucose uptake and glycolysis, but not glucose oxidation [141]. Consequently, glycolysis and glucose oxidation rates are mismatched, leading to glycolytic intermediates accumulation, such as glucose-6-phosphate, which regulates insulin-mediated and carbohydrate-mediated cell growth through mTORC1 activation [142, 143]. Moreover, increased accumulation of glycolytic intermediates improves both hexosamine biosynthetic pathway and pentose phosphate pathway, which in turn are involved in pathological hypertrophy development due to the biosynthesis of glycoproteins, protein O-GlcNacylation, and excessive accumulation of NADPH [141, 144, 145].

Finally, increased glucose reliance per se is not detrimental if the energetic demand is met in a healthy heart. However, in the long term, the metabolic remodelling coupled with increased glucose consumption could impair heart flexibility to use other substrates, thus promoting HF progression and onset [144].

### 3.2 Fructose Metabolism 

It has been reported an upregulation of the fructose metabolism in pathological hypertrophy. Hypoxia inducible factor-1α (HIf-1α) enhances the expression of splice factor 3b subunit 1 (*SF3B1*) and mediates ketohexokinase (KHK) pre-mRNA alternative splicing, which in turn increases cardiomyocytes fructose uptake through *SLC2A5* expression (encoding for GLUT5) and fructose to fructose-1-phosphate conversion. Fructose metabolites promote CH by upregulating both protein and lipid biosynthesis [146]. Moreover, in PO murine model, depletion of *SF3B1* leads to CH inhibition, whilst SF3B1 has been reported to be increased in patients affected by aortic stenosis and hypertrophic cardiomyopathy [146].

### 3.3 Ketone Body Metabolism

Ketone bodies represent a good alternative fuel source produced by liver mitochondria from fatty acids, due to several stimuli, such as exercise, fasting, ketogenic diet or untreated diabetes. In the heart, ketone bodies are metabolized into acetyl-CoA, which in turn is used for energy production tricarboxylic acid cycle and/or oxidative phosphorylation [147]. Sodium-glucose cotransporter 2 inhibitors (SGLT2i), a class of drugs recently approved for non-diabetic HF patients, enhance ketone bodies production, in particular β-hydroxybutyrate levels, through increased lipolysis. Increased ketogenesis benefits due to SGLT2i were reported in a nondiabetic pig model of HF. In this study, empagliflozin ameliorated the left ventricular remodelling and systolic function by improving the cardiac energy [148, 149]. Moreover, it was observed that β-hydroxybutyrate inhibits class I HDACs through histone acetylation and increases oxidative stress resistance [150]. Though, in a study on rat heart model, it was reported that ketone bodies can induce acute contractile dysfunction due to TCA cycle inhibition by sequestering CoA, which reverse with glucose or TCA-cycle intermediates use [151]. Moreover, mitochondrial proteins hyperacetylation were found to be increased in HF and it can be probably partially due to increased level of mitochondrial acetyl-CoA produced by chronic utilization of ketone bodies beyond TCA cycle acetyl-CoA saturation [152]. Therefore, further studies to better clarify the effective benefits of increased ketone levels against pathological hypertrophy are needed.

### 3.4 The Role of AMPK in Metabolic Reprogramming

Adenosine monophosphate-activated protein kinase (AMPK) regulates heart energy metabolism and is mainly activated by increased AMP and ATP depletion. AMPK main role is to enhance ATP production and reduce energy-consuming biosynthetic pathways by negatively regulating mTOR [153, 154]. ATP is the “molecular unit of currency” of intracellular energy transfer, and, when used during metabolic process, it is converted into AMP. During energy depletion, AMP levels are increased, and the binding and activation of AMPK is facilitated, through liver kinase B1, calcium/calmodulin-dependent protein kinase kinase 2 (CaMKK2), and TAK1, with glucose metabolism enhancement [155]. AMPK protein expression and activity is increased in human HF [156]. Several studies show that a pharmacological AMPK activation is associated with a reduced PO-induced hypertrophy [157, 158, 159], while mice with AMPK depleted activity experienced increased pathological hypertrophy and contractile dysfunction [160, 161, 162, 163]. However, results from rodent studies should be careful interpreted, also considering possible different regulatory mechanisms of AMPK cascade, also including isoform-specific function of AMPK across the species [156].

### 3.5 Mitochondrial Proteins Acetylation

Sirtuins (SIRT1–SIRT7) are a family of ${\mathrm{NAD}}^{+}$-dependent protein deacetylases that regulate cell survival, metabolism, and longevity. Mitochondrial proteins are hyperacetylated in both hypertrophy and HF, due to ${\mathrm{NAD}}^{+}$ levels reduction, with a consequent sirtuins inactivation [145, 152, 164]. Within the mitochondria, SIRT3 deacetylates several key metabolic enzymes: acetyl-coenzyme A synthetase, glutamate dehydrogenase, and subunits of electron transport chain, antioxidant proteins, and proteins involved in maintaining heart mitochondrial integrity [165, 166]. Moreover, in murine model it was demonstrated that SIRT3 activation through honokiol was able to block the development and can reverse pathological hypertrophy [167]. Furthermore, it is also reported that in PO mice nicotinamide mononucleotide supplementation can restore both cardiac function and energy metabolism [153, 168]. SIRT2 and SIRT6 were reported to be reduced in pathological hypertrophy, whereas their upregulation is associated with pathological hypertrophy protection [169]. SIRT1, instead, plays a double role. On the one hand SIRT1 serves as age-related cardiac pathological hypertrophy attenuator [170]. On the other hand, SIRT1 is involved in pathological hypertrophy worsening by suppressing ERR (estrogen-related receptor) target genes through PPARα interaction [171]. Finally, SIRT4 depletion is associated with angiotensin II-induced CH and fibrosis attenuation, whereas its overexpression with a worsening one [172].

Increased mitochondrial protein acetylation may contribute to impaired mitochondrial fuel oxidation and respiration, contributing to the vicious cycle of “energy starvation”, which in turn promotes HF development [152].

### 3.6 Heart and Other Organs Metabolic Crosstalk

It has been suggested the heart role in other organs metabolism, mainly through cardiokines secretion. In particular, cardiac miRNA208a and RNA polymerase II transcription subunit 13 (MED13) targets liver and white adipose tissue inducing metabolic gene expression upregulation and mitochondria increased number [173, 174]. Obese and diabetic patients show an increased pro-inflammatory adipokines expression, promoting a low-grade inflammation which is involved in the metabolic dysfunction onset [175]. In these settings, the anti-inflammatory adipokines, such as adiponectin which have proven to suppress PO-induced hypertrophy through cardiomyocytes AMPK activation, are reduced [160, 176]. Leptin is a hormone associated with white fat tissue, satiety process, and increases energy expenditure. In the heart it has been associated with morphological and functional alterations, with increasing cardiac muscle size and decreasing cardiac output [177]. In fact, it has been reported that leptin activated downstream proteins leads to rho-associated protein kinase (ROCK) activation, which in turn induces pathologic hypertrophy through several pathways (ERK1/2, MAPK and AKT/mTOR) [178].

It is also thought that adipose tissue may modulate pathological hypertrophy through exosomes secretion, though exosome research is still in its infancy [179, 180]. Finally, PPARγ agonists have proven to induce CH and HF development as an adverse effect in both human and murine model. In particular, a murine study has underlined that this issue is linked to cardiomyocyte-specific PPARγ deficiency, instead of the adipocyte-specific PPARγ deficiency [180]. It is thought that the underlying mechanism could be due to miR-200a adipocytes exosomes secretion, which in turn decrease TSC1 levels, thus activating mTOR hypertrophy protective pathway [180].

### 3.7 Metabolic Intermediates Accumulation and Diabetic Cardiomyopathy Development

Metabolic intermediates can accumulate in the heart, thus inducing cardiomyopathy. In obese, insulin resistant or diabetic patients a new entity called “diabetic cardiomyopathy”, also known as lipotoxic cardiomyopathy, has been described [180, 181, 182]. The key role of glycaemic control on the pathogenesis and outcome of coronary heart disease is well known [183, 184, 185]. Nevertheless, diabetic cardiomyopathy (DCM) is characterised by both hypertrophy and gradual HF with damaging cardiac remodelling, such as fibrosis and diastolic and systolic dysfunction, which is not directly related to coronary artery disease [186]. The main promoters of DCM are insulin resistance and subsequent hyperglycaemia, associated with altered fatty acid metabolism, altered calcium homeostasis and inflammation. Pro-inflammatory cytokines production (interleukin-6, tumour necrosis factor-α, monocyte chemoattractant protein-1 and nuclear factor-κB) triggers myocardial damage. Insulin resistance results in increased fatty acid oxidation and lipotoxicity, which further induce cardiac myocyte apoptosis and contractile dysfunction. Oxidative stress, advanced glycation end products and reduced coronary nitric oxide synthase affect the mechanisms of calcium homeostasis leading to its accumulation during diastole, increased cardiac stiffness and impaired relaxation, all of which result in contractility dysfunction. Dysregulation of many different miRNAs would appear to promote to the pathogenesis of cardiovascular diseases and are involved in diabetic cardiomyopathy development [187]. Actually, DCM represents only one of several chronic complications due to insulin resistance in the type 2 diabetic patient [188, 189, 190, 191, 192, 193, 194]. Diabetic cardiomyopathy’s hallmark is represented by intramyocardial lipid accumulation, which also appear to be associated with diastolic dysfunction [182, 195]. As reported in rodent studies, it seems that cardiomyocytes toxicity is not linked to triglycerides accumulation [196]. On the other hand, it has been suggested that ceramides, acylcarnitines, and diacylglycerol (DAG) accumulation, cellular compartmentalization, and storage are involved in several biological processes, in particular mitochondrial function and cellular metabolism, growth, and proliferation, though little is still known. Ceramides are lipid molecules mainly located on cell membranes, whereas their cytosolic accumulation has been reported to be associated with insulin resistance and apoptosis enhancement [197, 198, 199, 200]. Ceramide accumulation in the heart is lipotoxic, probably due to AKT reduced activity and increased fetal gene expression, and it can also lead to cardiomyocyte hypertrophy development [201, 202]. Moreover, in transgenic mice with cardiac-specific overexpression of glycosylphosphatidylinositol-anchored human lipoprotein lipase, pharmacological inhibition of ceramide biosynthesis has proven to improve diabetic cardiomyopathy [203]. Nevertheless, it is still debated the functional consequence of ceramides increased levels in diabetic cardiomyopathies [204]. Myocardium and serum of patients affected by HF have shown increased levels of both total and very long-chain ceramides, which were partially reversed after cardiac unloading [205]. In the same study, rodents following myocardial infarction showed increased level of both serine palmitoyl transferase (SPT), the rate-limiting enzyme of the de novo pathway of ceramide synthesis, and ceramides accumulation [205]. Pharmacological inhibition of SPT was associated with reduced ventricular remodelling, fibrosis, and macrophage content following myocardial infarction, whereas genetic deletion of *SPTLC2* preserved cardiac function following myocardial infarction [205, 206]. Moreover, *SPTLC1* and *SPTLC2* overexpression, such as during PO, was reported to enhance ceramide accumulation and apoptosis, and to reduce *in vitro* cardiomyocytes oxidative metabolism [205, 207].

Acylcarnitine production derives from long-chain fatty acids modification induced by acyl-CoA synthetases and by carnitine O-palmitoyltransferase 1, muscle isoform (CPT1M) in the cardiomyocytes’ outer mitochondrial membrane and is later carried into mitochondrial matrix through carnitine/acylcarnitine carrier protein, where it is metabolized in free carnitine and long-chain acyl-CoA. Heart acylcarnitine levels are increased in pathological hypertrophy setting, though it has been reported to be decreased 8 weeks after transverse aortic constriction and myocardial infarction [130, 208, 209]. In chronic HF patients, circulating long-chain acylcarnitine levels were independently associated with adverse clinical outcomes and, in end-stage disease, decreased after long-term mechanical circulatory support [210]. Nevertheless, the underlying mechanism leading to acylcarnitine level modification remains unknown.

Diacylglycerol, beyond being a lipid metabolite, is also a second messenger which induces insulin resistance by indirectly suppressing IRS1 phosphorylation, and inflammation through NF-κB activation in both diabetic patients and rodents [211, 212]. In HF patients, cardiac fatty acid content is reduced, though diacylglycerol levels are increased together with increased membrane PKC localization, and decreased AKT activity [211]. In end stage HF patients, it was shown that mechanical unloading with ventricular assist device implantation can correct diacylglycerol myocardial accumulation and lipotoxicity and modulate PKC and insulin–PI3K–AKT signalling, thus also underlying the correlation between diacylglycerol levels and insulin signalling [213]. Finally, diacylglycerol acyltransferase 1 depleted mice, an enzyme which convert diacylglycerol to triglycerides, are characterized by increased levels of diacylglycerol and ceramides, increased PKCα activation, along with reduced heart contractile function and survival [214].

DCM could recognise two different phenotypes based on different molecular adaptation and damage [215]. The first phenotype of DCM is related to increased systemic inflammation and characterised by concentric hypertrophy with preserved left ventricular diastolic and systolic function, increased myocardial stiffness and high left ventricular telediastolic pressure [215]. This isoform has several inflammatory/fibrosis biomarkers such as IL-1β, TNF-α, IL-18, IL-6, TGF-β and Galectin-3. Hypertrophy and interstitial fibrosis represent the final effects of the inflammatory cascade with the development of HFpEF. The second proposed DCM phenotype has the dilated pattern, characterised by eccentric remodelling of the left ventricle with reduced systolic function. HF with reduced ejection fraction (HFrEF) DCM results from cardiomyocyte cell death usually caused by ischaemic heart disease. Specifically, in HFrEF DCM, over-regulation of the free radical-producing enzyme nicotinamide adenine dinucleotide phosphate oxidase (NOX2) has been reported in cardiomyocytes secondary to ischaemic damage. Increased cardiomyocytes free fatty acids uptake in DM leads to mitochondrial dysfunction and increased expression of pro-apoptotic genes. Two other critical mechanisms associated with HFrEF DCM are fibrosis replacement and autoimmunity. Thus, lipotoxicity or NFkB activation by AGEs together with hyperglycaemia are the main players in fibrosis replacement, mainly due to fibroblasts PKC activity. In this sense, several biomarkers are higher in this phenotype. NTproBNP and high-sensitivity troponin T, biomarkers of myocardial wall stress and cardiomyocyte damage respectively, showed high accuracy in recognising HFrEF patients with left ventricular eccentric remodelling [216].

## 4. Conclusions

CH mainly develops in response to an increased preload and/or afterload and can evolve to cardiac impaired contractility, with a worsening outcome and increased social burden. Despite current large knowledge, most of which based on murine models, the pathophysiology of CH, as well as diabetic cardiomyopathy development and progression is still far from being fully explained.

More in-depth knowledge of both etiologic and pathogenic mechanisms is an exciting challenge for target-specific treatments development and to prevent HF onset.
